# *Actinobacillus ureae* may be a critical pathogen in patients with predispositions: A case report and review of the literature

**DOI:** 10.1097/MD.0000000000036087

**Published:** 2023-11-17

**Authors:** Qian Yang, Qiong Zhong, Bo Wu, XiaoDu Xu, WeiMin Li, BoHao Zhao, Min Luo, XingHua Zhu, Dan Ye, YanChun Huang

**Affiliations:** a Department of Laboratory Medicine, The First People’s Hospital of Longquanyi District, Chengdu/West China Longquan Hospital Sichuan University, Chengdu, China; b Department of Radiology, The First People’s Hospital of Longquanyi District, Chengdu/West China Longquan Hospital Sichuan University, Chengdu, China; c Department of Neurosurgery, The First People’s Hospital of Longquanyi District, Chengdu/West China Longquan Hospital Sichuan University, Chengdu, China.

**Keywords:** *Actinobacillus ureae*, case report, craniocerebral surgery, infection, pneumonia

## Abstract

**Rationale:**

*Actinobacillus ureae (A. ureae*) is an unusual commensal of human respiratory flora, rarely causing human infection. The predisposing factors, identification, clinical features, and antibiotic therapy of *A. ureae* are seldomly reported. Herein, we present a case of 64-year-old man affected by *A. ureae* pneumonia after intracranial surgery.

**Patient concerns and diagnoses:**

A 64-year-old male was admitted with vomiting, drowsiness, and a severe disturbance of consciousness and was later diagnosed with cerebral hemorrhage by computed tomography images. After a craniocerebral surgery, the patient suffered from intractable pneumonia, experiencing treatment failure with multiple anti-bacterial agents. Sputum culture yield pure colonies of *A. ureae*, confirmed by matrix-assisted laser desorption/ionization time of flight and 16S rRNA gene sequencing.

**Interventions:**

Minocycline (100 mg p.o. per 12 hours) with a course of 15 days was administrated for this patient.

**Outcomes:**

The respiratory symptoms, presenting as intermittent coughing with purulent and yellowish sputum, were gone. A 3-month follow-up examination showed a complete resolution of radiological findings.

**Lessons:**

Clinically, the actual incidence of *A. ureae* pneumonia may be higher than that we generally recognized, and clinicians should consider *A. ureae* as a possible etiologic agent in patients with predispositions. Currently, *A. ureae* may be susceptible to penicillin, ampicillin, and third-generation cephalosporins. Other antibacterial agents, such as tetracycline, amoxicillin/clavulanic acid, and aminoglycosides also respond well and can be a choice in the treatment of *A. ureae* infections.

## 1. Introduction

*Actinobacillus ureae (A. ureae*), a gram-negative bacillus, was first described and isolated from human noses cultures in 1960 as a variant of *Pasteurella haemolytica*, named as *Pasteurella haemolytica var. ureae*.^[1]^ Jones found this organism in the sputum of patients with chronic bronchitis and bronchiectasis, and further studies concluded that it represented a separate species named as *Pasteurella ureae (P. ureae*) in 1962.^[2]^ It was not until 1986 that a new classification based on DNA-DNA hybridization data was proposed by Mutters and his colleagues,^[3]^ that is, the transfer of *P. ureae* to the genus *Actinobacillus* under the combination *A. ureae*. It has been cultured from the respiratory tracts of patients with sinusitis, atrophic rhinitis, and chronic airway disease, as well as healthy individuals.^[4,5]^ This organism was usually harmless to humans and regarded as an uncommon commensal of the human respiratory flora. However, it can lead to serious infections in patients with underlying conditions, including skull fracture, alcoholism, or immune compromise.^[6–9]^

The first report of human infection appeared in 1961 when it was discovered from cerebrospinal fluid (CSF) cultures of a 39-year-old man who developed meningitis.^[6]^ It was evident from later reports that *A. ureae* was an etiologic agent of various diseases, including pneumonia,^[8]^ conjunctivitis,^[10,11]^ septic arthritis,^[9]^ bone marrow infection,^[12]^ spontaneous bacterial peritonitis,^[13]^ otitis media^[14]^ and so on. Over the decades there have been a few cases of infections caused by *A. ureae* in the literature, and even fewer cases reported of *A. ureae* pneumonia.

Here, we report a case of a Chinese man suffering from pneumonia due to *A. ureae*, and the literature on *A. ureae* is briefly reviewed. To the best of our knowledge, there has been no previous report of *A. ureae* as a causative organism in pneumonia from China.

## 2. Case presentation

Written informed consent was obtained from the patient and his wife for inclusion in this report. The Ethics Committee of The First People’s Hospital of Longquanyi District, Chengdu/West China Longquan Hospital Sichuan University approved the study.

A 64-year-old male, who had a past medical history of hypertension for more than 5 years without receiving orthodox treatment, was admitted to the emergency department with a 3-hour history of symptoms beginning with vomiting bloody gastric contents, followed by drowsiness, and progressing to a severe disturbance of consciousness, on August 27, 2021. The patient had a previous history of emphysema and bilateral bullae. He had neither seizures and fever nor cough and expectoration. There was no history of smoking, alcoholism, liver disease, tuberculosis, cranial traumas, and any remarkable family illness. At the emergency room, physical examination showed a body temperature of 36.6°C, pulse rate of 85 beats/min, respiratory rate of 17 beats/min, and blood pressure of 192/111 mm Hg. The rest of positive physical findings included a positive Babinski sign, neck stiffness, and a state of consciousness of superficial coma. The patient was unable to pronounce, and his eyes were in an unresponsive state to the light and pain, while he was responsive to the pain by limb withdrawal, thus, a Glasgow Coma Score of 6 was observed. Upon worsening consciousness, an emergent computed tomographic (CT) scan of the head was conducted, unveiling a severe intracerebral hemorrhage of the left-sided basal ganglia. The size of the hematoma was about 5.1 cm × 5.5 cm × 3.0 cm, with a volume approaching 50 mL. In a chest CT scan, the patient had bilateral bullae with emphysema, as well as mild inflammation in the lungs (see Figure S1A, Supplemental Digital Content, http://links.lww.com/MD/K770). Laboratory tests showed an elevated white blood cell (WBC) count of 20.03 × 10^9^/L with 88.3% neutrophils, and cefuroxime as a prophylactic antimicrobial agent (1.5 g i.v.gtt per 8 hours) was empirically administrated. Subsequently, a craniocerebral surgery of microscopical craniotomy hematoma removal, ventricle trepanation, and drainage was carried out within 4 hours of admission.

**Figure 1.**
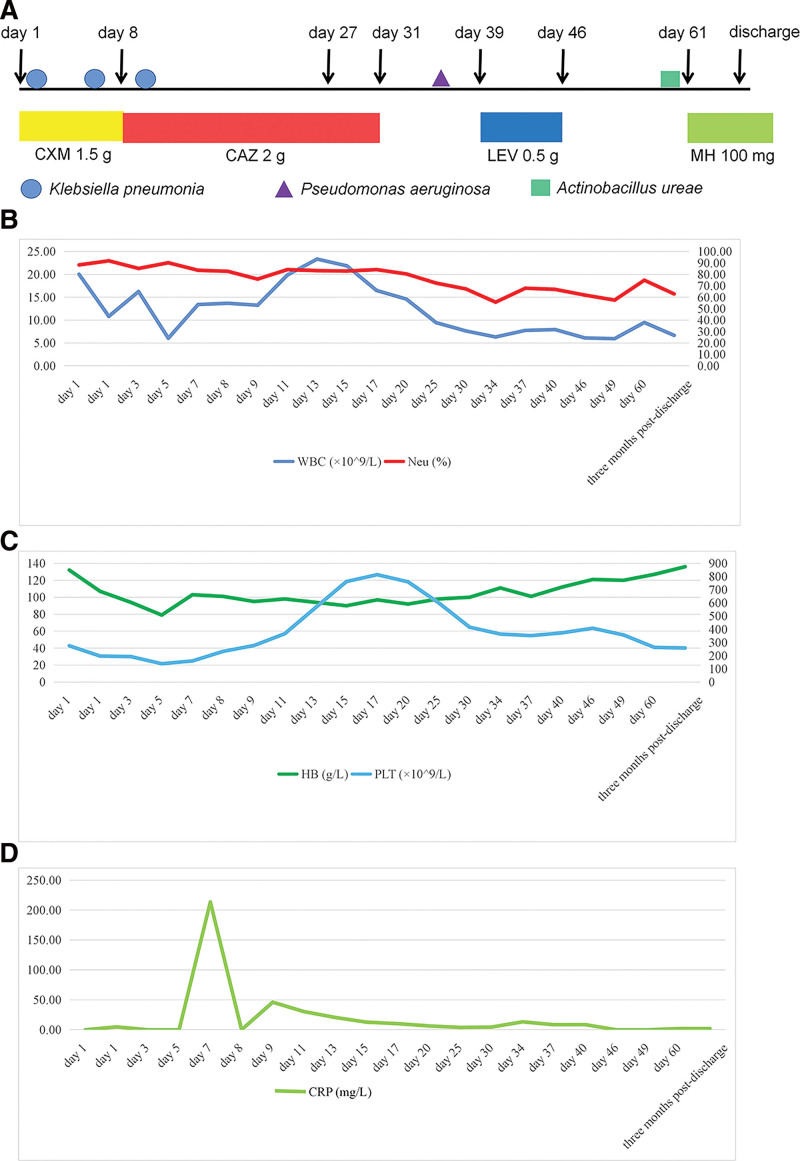
The clinical course, laboratory findings, and anti-infective therapy after hospitalization. (A) A brief timeline of the clinical course, including pathogens and antibiotic therapy. (B) Dynamic changes of WBC counts and neutrophil percentage at different time points, with reference ranges of 4–10 × 10^9^/L and 50–70%, respectively. (C) Dynamic changes of platelet counts and hemoglobin at different time points, with reference ranges of 100–300 × 10^9^/L and 130–175 g/L, respectively. (D) Dynamic changes of CRP during different periods with a reference range of 0–8 mg/L. The CRP was not performed on hospital day 3, day 5, day 8, day 46, and day 49.

**Figure 2.**
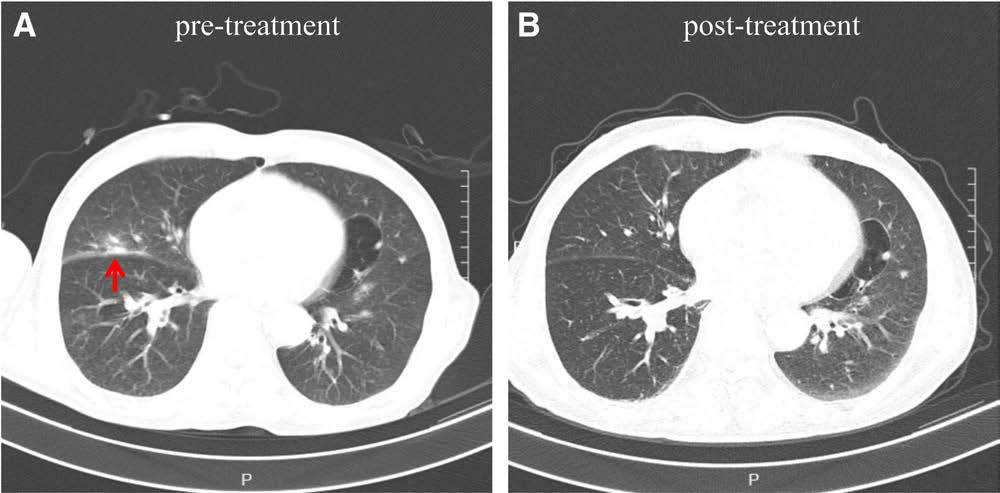
Chest CT scans during anti-infective therapy. (A) Radiographic abnormalities before antimicrobial treatment showed the inflammatory foci were noted in the left lung (red arrowhead). (B) Radiographic imaging after 3 months of treatment with minocycline, showed the inflammatory foci in the left lung was gone.

**Figure 3.**
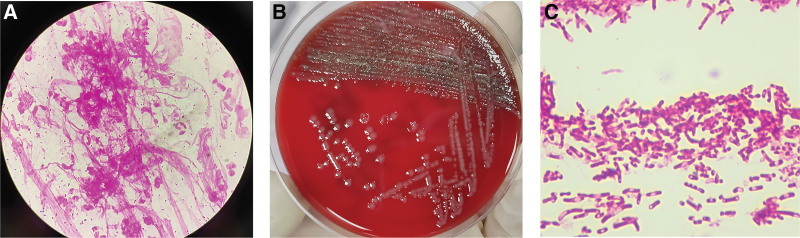
Morphological appearances of *A. ureae*. (A) A gram-stained smear of sputum under the microscope, showed many neutrophils, small gram-negative bacilli and coccobacilli, and the presence of mucus (10 × 100). (B) The pure growth of *A. ureae* on blood agar after incubation for 24 hours at 37°C in a CO_2_-enriched environment, showed nonhemolytic and translucent colonies. (C) Gram staining of colonies in Figure 3B revealed vacuolated gram-negative bacilli and bipolar staining (10 × 100).

This patient, with endotracheal intubation and mechanical ventilation, remained in a coma after the surgery. On hospital day 1, a high-quality sputum sample obtained after the administration of cefuroxime isolated *Klebsiella pneumoniae* (Fig. 1). Given the pulmonary infection, cefuroxime was continued (1.5 g i.v.gtt per 8 hours for 8 days). On hospital day 5, the patient had a fever, with a body temperature up to 38°C. Repeated sputum culture was sent to the clinical microbiology laboratory and had moderate growth of the same organism. Later in the day, he experienced sudden shortness of breath accompanied by a progressive decrease in oxygen saturation, then the tracheostomy with continuous positive airway pressure was immediately performed to improve hypoxemia and respiratory function. Over the next few days, recurrent attacks of fever continued, with body temperature ranging from 38.3°C to 39°C. Two sets of blood cultures were performed and yielded sterile after 5 days of incubation. On hospital day 8, he coughed with excessive phlegm, and moist rales were heard in the lungs. A repeated chest CT scan indicated bilateral, multiple inflammatory lesions, especially in the left lung (see Figure S1B, Supplemental Digital Content, http://links.lww.com/MD/K770); thus, the antibiotic was switched to ceftazidime. Since neither central fever nor intracranial infection could be ruled out, a lumbar puncture was conducted. Examination of the CSF showed 11 × 10^6^/L WBC counts with 86% neutrophils. The protein concentration and glucose concentration were both elevated, which were 3.62 g/L and 4.91 mmol/L, respectively. Culture and direct smear of the CSF were negative. Gradual improvement followed, and his temperature returned to normal. The state of consciousness recovered slowly and to some degree, he could respond to the doctor’s advice, such as opening his eyes and moving his fingers. On hospital day 31, ceftazidime was withdrawn due to clinical improvement (2 g i.v.gtt per 12 hours for 24 days). Shortly 5 days later, the cough returned, with slightly viscous and greenish-yellow sputum, and the isolate of sputum culture was later identified as *Pseudomonas aeruginosa*. On hospital day 36, another chest CT was performed, demonstrating scattered inflammatory foci and small bilateral pleural effusions (see Figure S1C and D, Supplemental Digital Content, http://links.lww.com/MD/K770). Based on the antimicrobial susceptibility testing, levofloxacin was given (0.5 g i.v.gtt per day for 8 days). Upon the state of consciousness gradually turning to clear, the tracheal intubation was removed on hospital day 54. Unfortunately, recurrent attacks of the respiratory symptoms occurred and continued, presenting as intermittent coughing with purulent and yellowish sputum on hospital day 60. A chest CT scan at that time revealed the inflammatory foci in the left lung (Fig. 2A). Gram stain of the sputum sample revealed many neutrophils, small gram-negative bacilli and coccobacilli, and the presence of mucus (Fig. 3A). The sputum culture yielded a heavy and pure growth of nonhemolytic translucent colonies on blood agar after incubation for 24 hours at 37°C in an atmosphere with 6.0–7.0% CO_2_ (Fig. 3B), while poor growth was present on chocolate agar and no growth observed on MacConkey agar (Fig. 3C). On staining of these colonies, vacuolated gram-negative bacilli and bipolar staining were partly exhibited. The organism was positive for oxidase and catalase. It was identified as *A. ureae* with a positive reaction for urease but reported as low discrimination by the gram-negative identification card on VITEK 2 Compact (bioMérieux, Lyon, France). Further identification was conducted by matrix-assisted laser desorption/ionization time of flight mass spectrometry (MALDI-TOF MS) Autof ms1000 system (Autobio Diagnostics, Zhengzhou, China). It was designated as *A. ureae* with a 9.335 confidence value. To validate identification from mass spectrometry, 16S rRNA gene sequencing was performed, and the isolates finally yielded *A. ureae* with 100% query coverage. The sequence of this case was submitted to NCBI (accession no. KX355773.1). Subsequently, minocycline was administered. The patient responded well to the treatment, followed by the relief of cough and purulent sputum. Two days later, a repeated sputum culture of *A. ureae* became negative.

On hospital day 65, he was discharged from the hospital and continued on a course of minocycline (100 mg p.o. per 12 hours for 15 days). One month after discharge, the patient remained well without cough and expectoration. Regrettably, he could not come to the hospital for blood testing and chest CT examination due to his incapability to walk. A 3-month follow-up examination showed a complete resolution of respiratory symptoms. Peripheral blood samples showed a WBC count of 6.66 × 10^9^/L with 62.9% neutrophils. A repeat of high-resolution chest CT showed the inflammatory foci in the left lung was gone (Fig. 2B) but remained aphasia and right-sided hemiplegia due to the hematoma of his left-sided basal ganglia before the operation.

Also, a computerized literature search of PubMed, Embase, the Cochrane Library, China National Knowledge Internet (CNKI), China Biology Medicine (CBM), and WanFang data was performed for relevant studies published until January 20, 2022. Medical subject heading terms (MeSH) and free-text words including “*Actinobacillus*,” “*Pasteurella*” and “*ureae*” were utilized to increase the sensitivity of the search. There were no language limits to publications. Additionally, we screened relevant references to identify potentially eligible reports. The study selection process is shown in Figure S2, Supplemental Digital Content, http://links.lww.com/MD/K771.

## 3. Discussion

*A. ureae*, formerly known as *P. ureae*, was first described by Henriksen and Jyssum in 1960.^[1]^ *A. ureae* is a commensal of the human upper respiratory tract, which may turn into an etiologic agent in the presence of predisposing conditions. To date, *A. ureae* has been identified as the principal pathogen in only 37 cases (Table 1). The majority of cases involved meningitis or meningoencephalitis,^[6,7,13,15–25]^ bacteremia,^[6,19,23–27]^ endocarditis,^[28–30]^ and respiratory infections,^[8,19,26,31,32]^ while bone marrow infection,^[12]^ conjunctivitis,^[10,11]^ septic arthritis,^[9]^ peritonitis,^[13]^ urinogenital infection^[33]^ and otitis media^[14]^ was also reported. Of these patients, men accounted for the majority (n = 30, 81%). The age of patients varied from 2-days to 75-years. 6 cases of death were reported, 5 of them died of serious infections, and only one pneumonia patient died of aspirating stomach contents. The mortality rate (n = 6, 17.1%) is relatively high based on the limited number of previous cases.

**Table 1:** *A. ureae* infections reported in the literature (37 cases).

Author	Year	Country	Gender, Age (yr)	Type of infection	Site of isolation	Underlying condition	Antibiotics	Antibiotic duration	Outcome
Frederiksen et al	1961	Denmark	M, 39	Meningitis	CSF	NA	NA	NA	Survived
Wang et al	1966	USA	M, 48	Meningitis	CSF	Chronic alcoholism skull fracture (17 d ago)	IV penicillin, chloramphenicol, oral sulfadiazine	3 wk	Survived
Thibault et al	1967	France	M, 16	Meningitis	CSF	NA	Penicillin, chloramphenicol, sulfisoxazole	NA	Died
Kolyvas et al	1978	Canada	M, 14	Meningoenc-ephalitis	CSF	Skull fracture (3.5 yr ago)	IV ampicillin	NA	Survived
Marriott et al	1983	Australia	M, 54	Meningitis	CSF	Alcohol abuse, schizophrenia, skull fractures	IV penicillin, then oral penicillin	4 wk	Survived
Brass et al	1983	USA	M, 40	Meningitis	CSF	Chronic schizophrenia, chronic alcohol abuse, periodontal disease	IV ampicillin, chloramphenicol	NA	Survived, vegetative state
Morlat et al	1989	France	M, 52	Meningitis	CSF	Chronic frontal sinusitis, previous skull fracture	IV ampicillin	2 wk	Survived
Kaka et al	1994	South Africa	M, 25	Meningitis	CSF	HIV, skull fracture	IV ceftriaxone, then penicillin	NA	Survived
Kingsland et al	1995	USA	M, 17	Meningitis	CSF	Skull fracture	IV penicillin, ceftazidime	NA	Survived
*Whitelaw et al	2002	South Africa	M, 22	Meningitis	CSF	Neurosurgery (6 yr ago) skull fracture (5 yr ago)	IV ceftriaxone	10 d	Survived
Yang et al	2009	China	F, 49	Meningitis	CSF	Hypertension, suffered a fall (1 yr ago)	IV ceftriaxone	NA	Survived
Bia et al	1978	USA	F, 53	Meningitis, bacteremia	CSF, blood	Neurosurgery (1 wk ago)	IV penicillin	12 d	Survived
Grewal et al	1983	the U.K.	M, 55	Meningitis, septicemia	CSF, blood	Diabetic	IV ampicillin	NA	Survived
Yagupsky et al	1985	Israel	M, 6	Meningitis, bacteremia	CSF, blood	Skull fracture (3 mo ago)	IV ampicillin, chloramphenicol	2 wk	Survived
Verhaegen et al	1988	Belgium	M, 26	Meningitis, bacteremia	CSF, blood	*Streptococcus pneumoniae* meningitis (10 and 8 yr ago), alcoholism, a heavy smoker	IV cefotaxime, penicillin	2 wk	Survived
Nathalie et al	2007	France	M, 75	Meningitis, bacteremia	CSF, blood	Waldenstrom’s macroglobulinaemia, *Streptococcus pneumoniae* meningitis (30 yr ago), head trauma, hypertension, alcohol abuse	IV cefotaxime then oral amoxicillin	3 wk	Survived
Gatti et al	1968	Congo	M, 2	Bacteremia	Blood	Malnutrition, hepatitis	NA	NA	Died
Maritz et al	1981	South Africa	M, 19	Septicemia bronchopne-umonia	Blood	Chronic active hepatitis, cirrhosis	IV gentamicin, cephalothin, metronidazole	NA	Died
Berardi et al	1984	France	M, 47	Septicemia	Blood	Liver cirrhosis, sinusitis, alcoholism	IV ampicillin, then oral clamoxyl	1 mo	Died
Yamamoto et al	1993	Japan	M, 59	Endocarditis	Blood	*Staphylococcus aureus* endocarditis (7 yr ago), a periodontal operation (2 wk ago)	IV piperacillin, gentamicin, then oral cefotiam	>6 wk	Survived
Zhao et al	2011	China	M, 45	Endocarditis	Blood	Heroin abuse (2 yr), methadone (1 yr)	NA	NA	Died
Xu et al	2016	China	F, 25	Endocarditis	Blood, bone marrow	NA	IV pazufloxacin mesilate, then meropenem, enoxacin	>40 d	Survived, superfici-al coma
Avlami et al	1997	Greece	M, 65	Bone marrow infection	Bone marrow	Rheumatoid arthritis, a heavy smoker, a mild drinker	IV benzylpenicillin, then oral tetracycline	4 wk	Survived
Starkebaum et al	1977	USA	M, 57	Pneumonia	Sputum	Alcoholism, emphysema, chronic organic brain syndrome, hip and rib fracture	Cephalothin	NA	Died
Yoshizaki et al	1981	Japan	M, 72	Bronchopne-umonia	Sputum	Chronic bronchitis	NA	NA	NA
Vay et al	1995	Spain	NA	Chronic bronchitis	NA	NA	NA	NA	NA
Perez et al	2000	Spain	M, 28	Pneumonia	Sputum	AIDS, IDU, HCV hepatitis	IV ceftriaxone	10 d	Survived
*Dawar et al	2016	India	M, 62	Pneumonia	Sputum	Bronchial asthma, oral corticosteroid, dilated cardiomyopathy	Amoxicillin/clavulanic acid	2 wk	Survived
Bogaerts et al	1985	NA	2 d	Conjunctivitis	Purulent discharge	NA	1% chloramphenicol ointment	NA	Survived
Ergin et al	2007	Turkey	F, 4	Acute conjunctivitis	Conjunctival scrub sampling	NA	Amoxicillin/clavulanic acid	5 d	Survived
Noble et al	1987	USA	M, 44	Spontaneous bacterial peritonitis	Blood	Liver disease, alcoholism	IV ampicillin, clindamycin, gentamicin	10 d	Survived
Bigel et al	1988	France	M, 2	Otitis media	Tympanic pus	Severe iron deficiency anemia	Oral amoxicillin/clavulanic acid	10 d	Survived
Kaur et al	2004	USA	F, 59	Multifocal septic arthritis	Joint cavity pus	Rheumatoid arthritis, etanercept, methotrexate	IV cefazolin, ciprofloxacin, then IV piperacillin/tazobacta-m and ciprofloxacin, oral ciprofloxacin	6 wk	Survived
Zhao et al	2008	China	M, 35	Urinogenital infection	Secretions	NA	Ciprofloxacin	NA	Survived
Zhao et al	2008	China	M, 28	Urinogenital infection	Secretions	NA	Ciprofloxacin	NA	Survived
Zhao et al	2008	China	M, 30	Urinogenital infection	Secretions	NA	Ciprofloxacin	NA	Survived
Zhao et al	2008	China	M, 33	Urinogenital infection	Secretions	NA	Ciprofloxacin	NA	Survived

CSF = cerebrospinal fluid, F = female, IV = intravenous, M = male, NA = not applied.

* Identified by 16S rRNA gene sequencing.

In most cases of *A. ureae* meningitis, a prior skull fracture, neurosurgery, and chronic alcohol abuse were observed, as well as the underlying diseases including diabetes, hypertension, and schizophrenia. We speculated that patients with chronic alcoholism, skull injury, and immune compromise were at increased risk of *A. ureae* infection. These indicated *A. ureae* meningitis should be suspected after a head injury or intracranial surgery especially when immune dysfunction existed, as appeared in a HIV-positive male who suffered serious meningitis.^[7]^ Similarly, alcoholism, chronic liver diseases, and immunocompromised conditions, such as receiving anti-TNF or corticosteroid therapy were also observed in non-intracranial infections.^[9,32]^ Interestingly, among the 6 reported cases of respiratory infections due to *A. ureae*, 3 occurred in patients with chronic lung disease: 1 patient with emphysema,^[8]^ 1 with chronic bronchitis,^[31]^ and another with bronchial asthma.^[32]^ As was the case with the patient described here, our patient had a previous history of emphysema and bilateral bullae. He remained to be bedridden for more than 1 month after intracranial surgery. Old age and male sex, the predisposing conditions mentioned in the earlier study,^[34]^ plus the multiple antibiotic therapy in our patient, might make this commensal organism into an etiologic agent.

Identification of *A. ureae* was based on the conventional manual methods including the morphology and biochemical characteristics, followed by commercial or automated systems, recently MALDI-TOF MS and molecular biology-based identification techniques such as 16S rRNA gene sequencing. As early as 1992, 16S rRNA gene sequencing was carried out to confirm the identity of the organism,^[35]^ while traditional phenotypic testing may cause improper identification, particularly when the isolate is rare, or the biochemical profile of the pathogen is uncommon, or even the automated systems do not have the panels of the organism. As described in the case of *A. suis* or *A. equuli* sepsis and meningitis,^[36]^ the isolates yielded *A. ureae* by the VITEK 2 Compact, while *A. suis or A. equuli* was suspected based on MALDI-TOF MS and finally confirmed by gene sequencing, which posed a question of the accuracy of identification in previously reported infections by *A. ureae*. In our case, we not only conducted traditional phenotypic testing but also performed MALDI-TOF MS and 16S rRNA sequencing, proving the identification achieved high reliability. However, there were 2 strains confirmed by 16S rRNA sequencing in all previous reports^[21,32]^ and only 1 strain was identified by MALDI-TOF MS.^[32]^ Compared with gene sequencing, the MALDI-TOF MS is more common in clinical laboratories due to the advantages of being quicker and cheaper, however, it also probably makes an erroneous identification. Here, we would like to highlight the importance of molecular techniques. Although gene sequencing seems to be unlikely to replace traditional phenotypic testing in the clinic, it can be considered an alternative when equivocal phenotypical identification existed. To the most, only by having a good knowledge of this organism can we culture, identify, and further analyze it to provide our patients a speedy and full recovery.

Interestingly, of the 37 reported cases of *A. ureae* infections, patients with meningitis, endocarditis, bacteremia, bone marrow infection, and spontaneous peritonitis all developed fever during the clinical course, and their cultures were obtained from sterile body fluids such as CSF, blood, and bone marrow. In most cases, there was an increase in WBC counts and neutrophils, and some were accompanied by a shift to the left. On the contrary, other patients, such as conjunctivitis and otitis media, did not experience fever. In this report, our patient encountered recurrent attacks of fever after a craniocerebral surgery, which lasted for several days and finally, the body temperature returned to normal. The inflammatory indexes, including WBC counts, the proportion of neutrophils, and C-reactive protein, did not show a remarkable increase. Therefore, we concluded that *A. ureae*, as an opportunistic pathogen, is absent of strong pathogenicity, but can lead to serious infections after its reproduction in humans, especially these sterile areas. Combined with the previously limited literature,^[8,31,32]^ there were no specific clinical features of *A. ureae* pneumonia observed, as well as physical examination. Patients with *A. ureae* pneumonia may or may not have a fever, with or without the increase of WBC, neutrophil percentage, and C-reactive protein. Radiographic presentations of *A. ureae* pneumonia are not specific either, but significantly, a tree-in-bud pattern on chest CT was observed by Dawar.^[32]^

Currently, there is no consensus on the antibiotics and treatment course of *A. ureae*. As depicted in previous clinical reports, *A. ureae* is susceptible to most β-lactam antibiotics, including penicillin, ampicillin, and third-generation cephalosporins like ceftriaxone, especially in patients with *A. ureae* meningitis. These antibiotic agents above are of favorable permeability into the blood-brain barrier. Other antibacterial agents, such as tetracycline, amoxicillin/clavulanic acid, and aminoglycosides also respond well and could be a choice in the treatment of *A. ureae* infections. In this case, since our patient had an administration history of third-generation cephalosporins and quinolones, minocycline was administrated for avoiding the potential resistance of these drugs. The susceptibility testing of *A. ureae* was not conducted due to limited laboratory conditions and a positive response to minocycline. Since a relatively high prevalence of antibiotic use has been reported in our country,^[37]^ prompt selection of appropriate empiric antibiotics has become increasingly important and difficult. Given all that, with timely identification and adequate therapy, previous reports indicated a relatively favorable outcome.

However, there is some insufficiency in the treatment of this case. The patient experienced treatment failure with multiple anti-bacterial agents, minocycline was empirically administrated without the susceptibility testing due to the limited laboratory conditions. Meanwhile, the duration of minocycline was only based on the patient’s clinical improvement. Clinical failure to monitor inflammatory indicators in a timely manner to evaluate the efficacy of antimicrobial therapy during the treatment. Fortunately, a 3-month follow-up examination confirmed a favorable outcome.

In conclusion, a 64-year-old man developed *A. ureae* pneumonia after intracranial surgery. Although rare, *A. ureae* should be considered as an opportunistic pathogen in the presence of underlying predispositions. A high index of suspicion and early identification, as well as appropriate antibiotic therapy, are important factors in a favorable outcome.

## Author contributions

**Conceptualization:** YanChun Huang.

**Data curation:** Qian Yang, Qiong Zhong.

**Formal analysis:** Qian Yang, Qiong Zhong, Bo Wu.

**Investigation:** Bo Wu, XiaoDu Xu, WeiMin Li, BoHao Zhao, Min Luo, XingHua Zhu, Dan Ye.

**Writing – original draft:** Qian Yang.

**Writing – review & editing:** YanChun Huang.

## Supplementary Material




