# Supplementary figures and images for: Actinobacillus ureae may be a critical pathogen in patients with predispositions: A case report and review of the literature
Source: Medicine (Baltimore). 2023 Nov 17;102(46):e36087. doi: 10.1097/MD.0000000000036087 (PMC10659659; doi:10.1097/MD.0000000000036087)

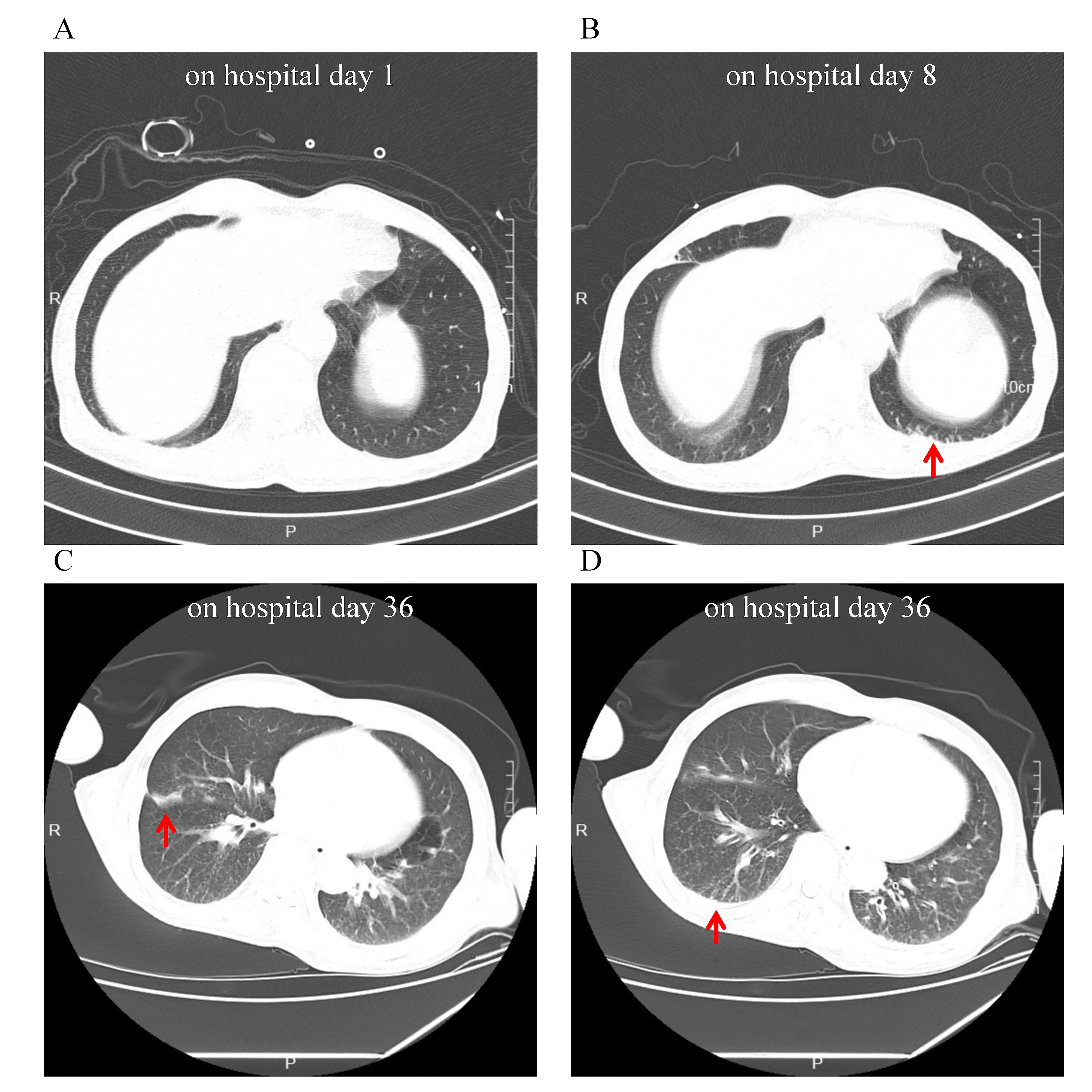

Supplement: Supplementary file 1 [file medi-102-e36087-s001.tif]

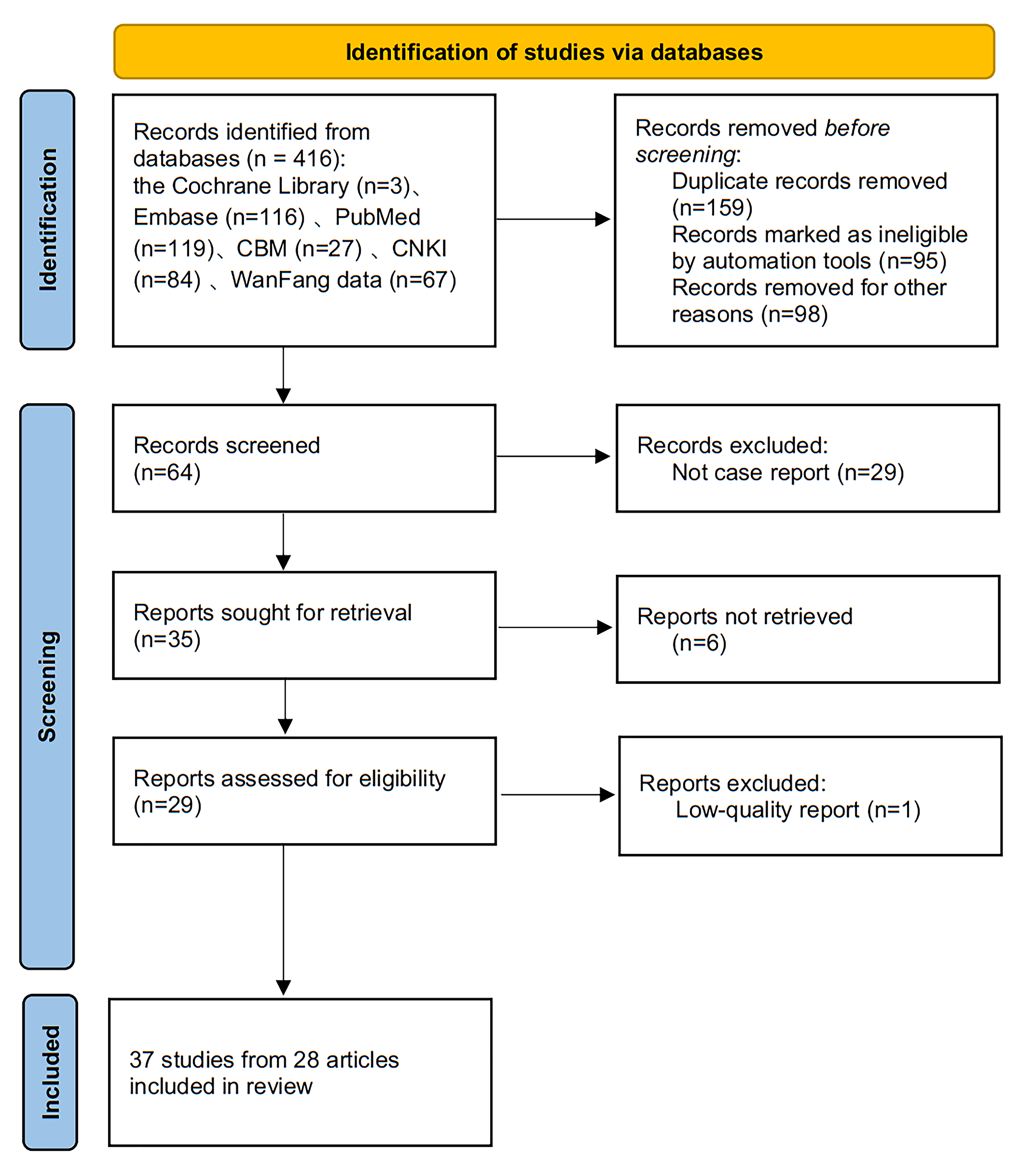

Supplement: Supplementary file 2 [file medi-102-e36087-s002.tif]
